# Omental Infarction Presenting as Right Iliac Fossa Pain: A Rare Radiological Diagnosis

**DOI:** 10.7759/cureus.95935

**Published:** 2025-11-02

**Authors:** Anoop Sunny, Ali Javaid

**Affiliations:** 1 General Surgery, Tameside General Hospital, Manchester, GBR; 2 General Surgery, Tameside and Glossop Integrated Care NHS Foundation Trust, Manchester, GBR

**Keywords:** abdominal imaging, case report, diagnosis of rare cases, general surgery and related subject, omental infarction, radiology, right iliac fossa pain

## Abstract

Omental infarction is a rare, benign cause of acute abdominal pain that frequently mimics appendicitis. Its recognition via imaging is essential to avoid unnecessary surgical intervention and guide evidence-based conservative management.

This report aims to emphasize the diagnostic role of contrast-enhanced computed tomography (CT) in differentiating omental infarction from appendicitis, which is essential to avoid unnecessary surgical intervention and guide management.

We report a case of a 40-year-old obese man who presented with acute right iliac fossa pain, clinically suggestive of appendicitis. Laboratory tests revealed normal inflammatory markers (white cell count = 5.6 × 10⁹/L; C-reactive protein = 9 mg/L). Contrast-enhanced CT demonstrated a 5 × 4 cm heterogeneous fatty lesion with surrounding inflammatory fat stranding, peritoneal thickening, and mild vessel engorgement in the right iliac fossa, with a normal appendix, findings consistent with primary omental infarction. The patient was managed conservatively with analgesics and anti-inflammatory medication, resulting in complete symptom resolution within five days.

CT remains the diagnostic gold standard for omental infarction, typically showing an ovoid fatty mass with hyperattenuating streaks and a preserved appendix. Differentiation from appendicitis is crucial to guide non-operative management.

This case highlights the importance of prompt CT evaluation in patients with atypical right iliac fossa pain. Recognizing the radiological features of omental infarction allows evidence-based conservative treatment, prevents unnecessary appendectomy, and reinforces the value of imaging-guided decision-making in acute abdomen.

## Introduction

Omental infarction is an uncommon cause of acute abdominal pain, accounting for fewer than four cases per 1,000 presentations initially suspected as appendicitis [1,2]. It typically affects the right side of the abdomen, where the greater omentum is longer and more mobile [3]. The condition is often misdiagnosed as appendicitis or cholecystitis due to overlapping clinical features such as localized tenderness and guarding [4,5].

Pathophysiologically, omental infarction results from compromised blood flow to a segment of the omentum, usually secondary to torsion or spontaneous venous thrombosis [1,5]. Predisposing factors include obesity, sudden body movements, trauma, and vascular anomalies [2,5]. Distinguishing between primary and secondary omental infarction is clinically important, as it influences management decisions and prognosis.

Computed tomography (CT) is the gold standard for diagnosis, revealing a well-circumscribed fatty lesion with hyperattenuating streaks and absence of appendiceal inflammation [1,6]. Ultrasonography, though sometimes used, has limited sensitivity and specificity [7]. Conservative management with analgesics and anti-inflammatory medication is generally effective [8,9].

This case is presented to emphasize the diagnostic role of CT in distinguishing omental infarction from appendicitis, particularly in obese patients presenting with right iliac fossa pain, and to highlight the favorable outcomes of conservative treatment.

## Case presentation

A 40-year-old man presented to the emergency department with a one-day history of severe, sharp right iliac fossa pain. The pain was constant, non-radiating, and worsened with movement. He reported mild nausea and vomiting but denied fever, bowel, or urinary symptoms.

His medical history included type 2 diabetes mellitus and prior investigation for non-specific cardiac chest pain. He had a body mass index (BMI) of 32.7 kg/m². On examination, he was afebrile (36.8°C) with localized tenderness, guarding, and mild rigidity in the right iliac fossa, without rebound tenderness or peritonism. Bowel sounds were present and normal.

Blood investigations (Table 1) showed a normal white cell count and mildly elevated C-reactive protein, consistent with a non-inflammatory process. Lactate and urinalysis results were within normal limits.

**Table 1:** Laboratory results at presentation.

Parameter	Result	Reference range	Unit
Hemoglobin	118	130–170	g/L
White cell count (WCC)	5.6	4.0–11.0	×10⁹/L
Neutrophils	3.5	2.0–7.5	×10⁹/L
C-reactive protein (CRP)	9	<5	mg/L
Lactate	1.2	0.5–2.2	mmol/L
Urinalysis	Normal	—	—

**Figure 1:**
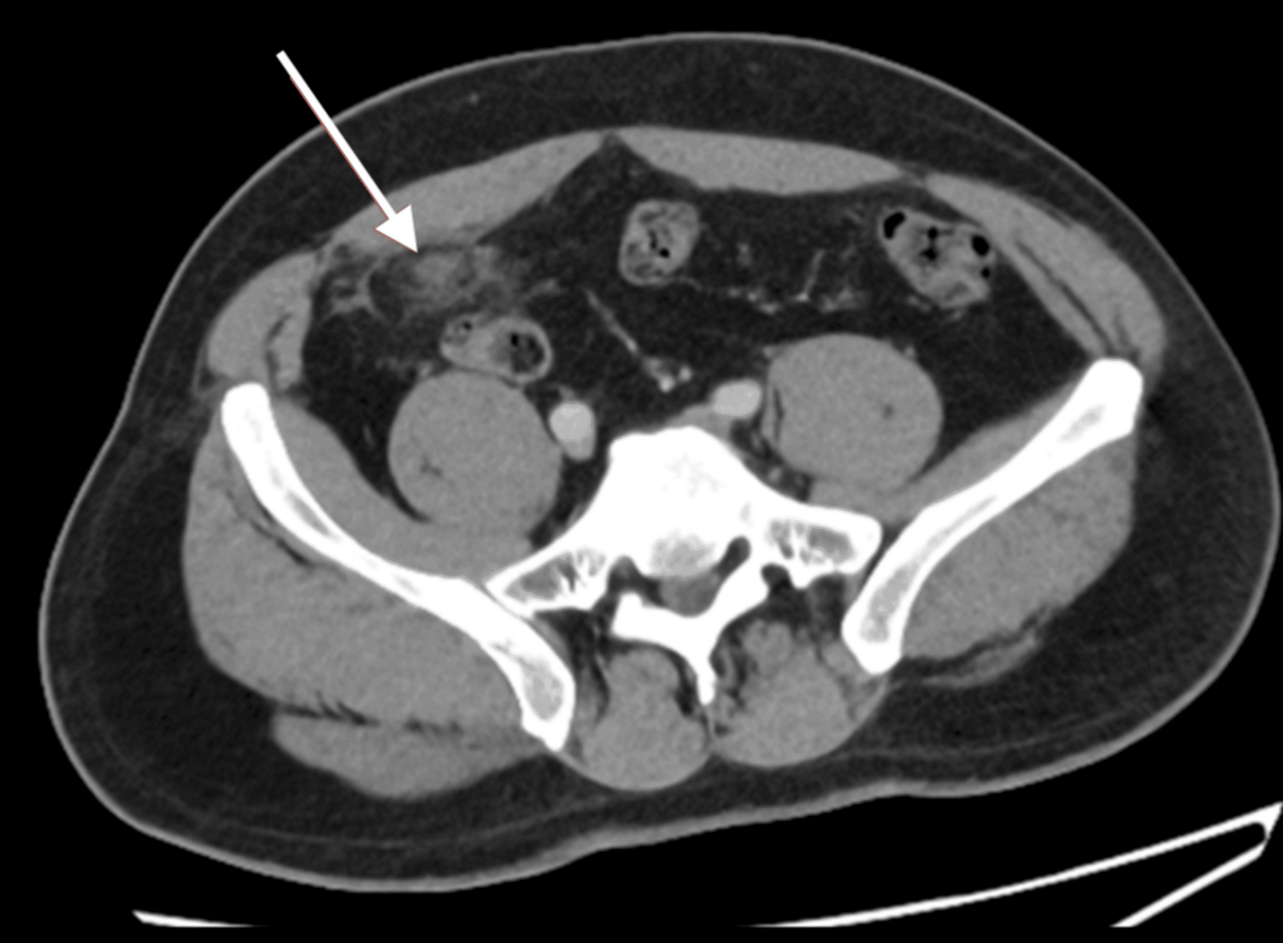
Axial CT image showing a focal area of fat with hyperattenuating streaks and surrounding stranding (arrow), with associated peritoneal thickening and mild vessel dilation.

**Figure 2:**
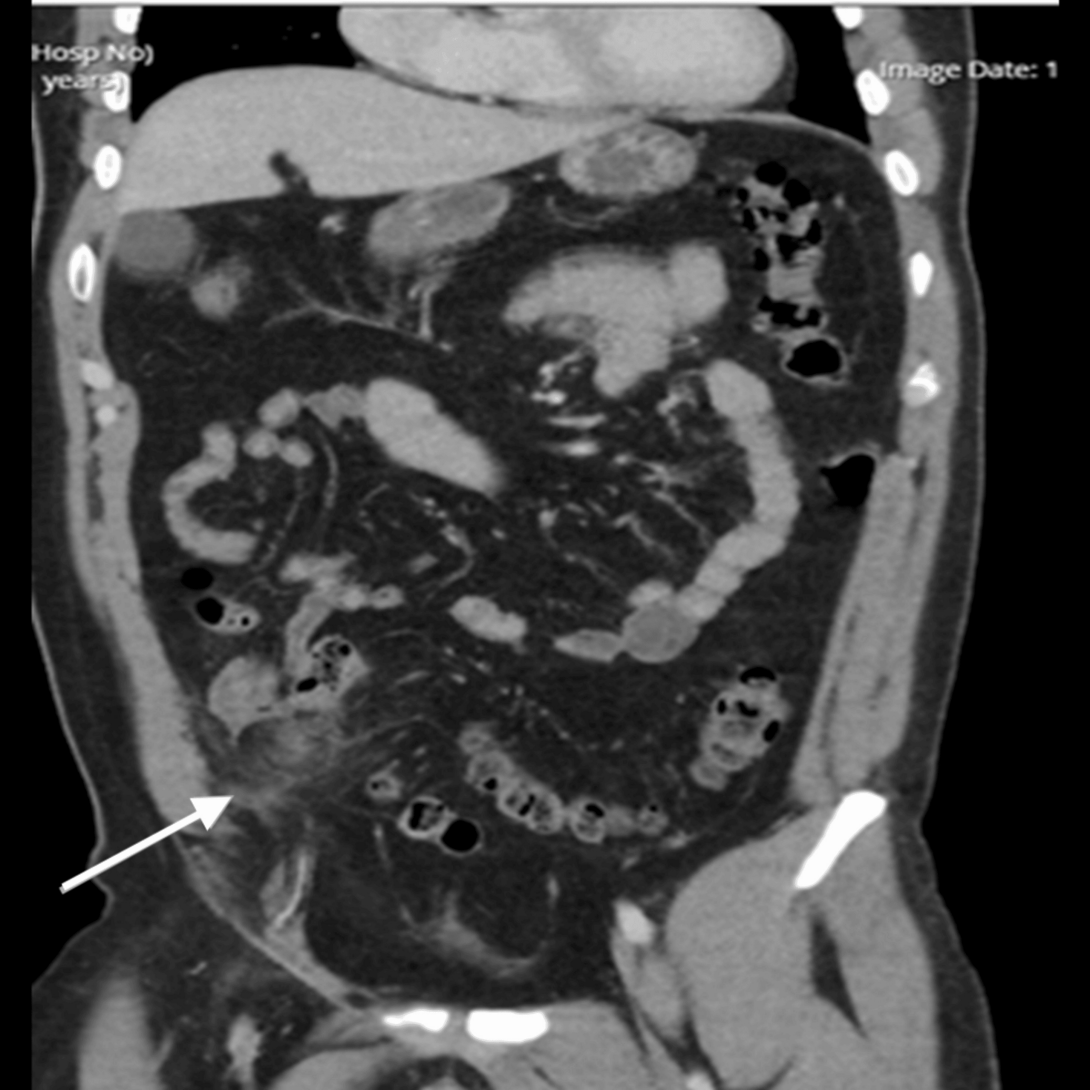
Coronal CT image demonstrating the same lesion in the right iliac fossa, with preserved appendix.

An initial differential diagnosis of appendicitis [1,3] was considered. Contrast-enhanced CT of the abdomen and pelvis revealed a 5 × 4 cm ovoid area of heterogeneous fat attenuation with surrounding inflammatory stranding, peritoneal thickening, and mild vascular engorgement in the right iliac fossa. The appendix appeared normal, with no periappendiceal inflammation or free fluid (Figures 1, 2).

Based on the CT findings consistent with primary omental infarction and the absence of peritonitis, conservative management was pursued [2,8]. The patient received oral analgesics (paracetamol 1 g every six hours, tramadol 50 mg every six hours) and antiemetics as required. His symptoms improved markedly within five days. He was discharged with outpatient follow-up and reported full recovery without recurrence at two weeks.

## Discussion

Omental infarction is a rare but important differential diagnosis in patients with right iliac fossa pain. The condition arises from torsion or venous thrombosis of the omental vessels, resulting in ischemia and localized fat necrosis [1,9]. Primary infarction typically occurs without an underlying cause, whereas secondary infarction may arise from intra-abdominal inflammation, hernias, or adhesions [5].

Predisposing factors include obesity, sudden body movements, trauma, and hypercoagulable states [2]. In our patient, obesity likely contributed to venous stasis within the omental vasculature.

CT is the imaging modality of choice [1,4]. Typical CT findings include a focal, ovoid fatty lesion ranging from 3 to 15 cm in size, exhibiting heterogeneous attenuation. Hyperattenuating streaks, often referred to as the “whirl sign,” are indicative of twisted omental vessels. Surrounding fat stranding and mild peritoneal thickening are commonly observed [1,6]. These features differentiate omental infarction from epiploic appendagitis [6,9] (smaller, pericolic fat lesion with a central hyperdense dot) and appendicitis [1,3] (inflamed appendix with periappendiceal fat stranding).

Most uncomplicated cases resolve spontaneously with conservative therapy, including analgesics and anti-inflammatory medications [8,9]. Surgical intervention is reserved for complications such as abscess formation [4,5], necrosis, or diagnostic uncertainty [6,10]. Conservative management is supported by case series showing full recovery within one to two weeks and no recurrence [2,9].

Unlike previously reported cases requiring surgical exploration [5,6], our patient was successfully treated conservatively with complete recovery, underscoring the diagnostic value of CT and the safety of non-operative management.

This case reinforces that prompt CT evaluation of atypical right iliac fossa pain can accurately diagnose omental infarction, preventing unnecessary appendectomy. In obese patients with normal inflammatory markers and localized tenderness, radiological assessment should be prioritized.

## Conclusions

Omental infarction, though rare, should be considered in the differential diagnosis of right iliac fossa pain. Its recognition is essential to prevent misdiagnosis as appendicitis and to guide appropriate management. CT remains the gold standard for diagnosis, with characteristic findings of a localized fatty lesion, fat stranding, and a normal appendix. Awareness of these imaging features allows clinicians to adopt conservative, evidence-based treatment, minimizing surgical risk and promoting rapid recovery.
